# A randomized, controlled cross-over trial of dermally-applied lavender (*Lavandula angustifolia*) oil as a treatment of agitated behaviour in dementia

**DOI:** 10.1186/1472-6882-13-315

**Published:** 2013-11-13

**Authors:** Daniel W O’Connor, Barbara Eppingstall, John Taffe, Eva S van der Ploeg

**Affiliations:** 1 School of Psychology and Psychiatry, Monash University, Kingston Centre, Warrigal Road, Cheltenham, Victoria 3192, Australia

**Keywords:** Lavender, Dementia, Behaviour, Mood, Randomised controlled trial

## Abstract

**Background:**

Lavender essential oil shows evidence of sedative properties in neurophysiological and animal studies but clinical trials of its effectiveness as a treatment of agitation in people with dementia have shown mixed results. Study methods have varied widely, however, making comparisons hazardous. To help remedy previous methodological shortcomings, we delivered high grade lavender oil in specified amounts to nursing home residents whose agitated behaviours were recorded objectively.

**Methods:**

64 nursing home residents with frequent physically agitated behaviours were entered into a randomized, single-blind cross-over trial of dermally-applied, neurophysiologically active, high purity 30% lavender oil versus an inactive control oil. A blinded observer counted the presence or absence of target behaviours and rated participants’ predominant affect during each minute for 30 minutes prior to exposure and for 60 minutes afterwards.

**Results:**

Lavender oil did not prove superior to the control oil in reducing the frequency of physically agitated behaviours or in improving participants’ affect.

**Conclusions:**

Studies of essential oils are constrained by their variable formulations and uncertain pharmacokinetics and so optimal dosing and delivery regimens remain speculative. Notwithstanding this, topically delivered, high strength, pure lavender oil had no discernible effect on affect and behaviour in a well-defined clinical sample.

**Trial registration:**

Australian and New Zealand Clinical Trials Registry (ACTRN 12609000569202)

## Background

The fragrant oil extracted from lavender (*Lavandula*) is used as a relaxant in many aged care facilities. While evidence in support of its use is mixed, its potential for sedative and anticonvulsant activity is clear. After rapid absorption via skin or respiration [1], lavender and its principal components, linalool and linalyl acetate, enter the brain where they act as depressants, inhibiting glutamate and GABA receptor binding [2,3].

From a behavioural perspective, lavender reduces conflict between mice; dampens their motor behaviour, and counters the stimulating effects of caffeine [4,5]. Its sedative properties are significant with 3% inspired linalool reducing spontaneous motor activity to a greater degree than 1 mg/kg of parenteral diazepam [6]. In humans, lavender has been shown to lower plasma cortisol levels [7,8]; reduce the need for post-operative analgesia [9], and exert anti-anxiety effects equivalent to those of lorazepam 0.5 mg daily [10]. It has no known adverse effects in customary, and even substantial, doses [11].

In an effort to find alternatives to psychotropic medications, there have been several controlled trials of lavender oil as a treatment of agitated behaviours in nursing home residents with dementia. Two trials had promising results. In the first, Holmes *et al*. sprayed a dementia ward with either 2% lavender oil or water for two hours daily on alternating days [12]. All 15 participants had severe dementia and daily agitation. When an observer wearing a nose clip rated participants’ behaviours on the Pittsburgh Agitation Scale, scores were 20% lower when exposed to lavender compared to water. In the second study, Lin *et al*. administered lavender or sunflower oils by vapour for an hour each night to 70 nursing home residents with marked dementia and agitation [13]. After the oils were administered in random order for three week periods, scores on the Cohen Mansfield Agitation Inventory (CMAI) fell by 7% with lavender compared to 1% with sunflower oil.

Four other controlled trials were less positive. Snow *et al*. pinned sachets infused with lavender, thyme or neutral oils for several hours daily to the collars of seven dementia unit residents [14]. Treatments were rotated every two weeks while nurses completed the CMAI on alternate days. There were no significant changes in scores with either lavender or thyme oil. Smallwood *et al*. randomly allocated 21 severely demented hospital patients to a lavender oil massage, neutral oil massage or vaporised lavender oil coupled with conversation [15]. Treatments were administered twice weekly at different times of day for unspecified periods. Videotapes made four times daily revealed no significant differences in behaviour. Gray and Clair studied 13 confused nursing home residents who were reluctant to swallow medications [16]. They were randomly rotated through exposures on four occasions each to lavender oil, sweet orange oil, tea tree oil and no aroma. Cotton balls infused with the oils were pinned to subjects’ collars 20 minutes before their medications were dispensed. Videotapes of residents’ responses to medication administration showed no significant differences in behaviour during any condition. Finally, Fujii *et al*. found no changes in blinded behaviour ratings after lavender oil was applied three times daily over a four week period to the collars of 14 out of 28 long-term patients with dementia [17].

Differences in lavender formulation, delivery methods, participant numbers, selection criteria, outcome measures and time frames make it almost impossible to compare reports [18]. It is not clear, therefore, why lavender has proved effective in two earlier studies but not in four others. Such a varied evidence base concerning a safe, inexpensive and readily implemented treatment makes it important to conduct further trials using optimal research methods [18].

Ideally, studies in this field require that: (i) participants demonstrate one or more behavioral symptom to a specified degree and frequency; (ii) treatments are deployed in time frames and settings when the behavioural symptom is most evident; (iii) behaviours are counted directly to limit the bias inherent in staff-completed rating scales; (iv) observers are blinded as much as possible, (v) and allowance is made for non-specific placebo effects [19]. For studies involving fragrance, researchers should wear nose clips or masks to maintain blinding and the control condition should involve a similar amount of time and physical contact with participants [20].

Optimal dosing regimens and formulations have yet to be established [20]. When lavender oil is diffused via droplets or nebuliser, as happened in five of the six previous studies, the amounts inhaled by an individual nursing home resident are likely to vary according to dispersion rates, room size, ventilation patterns and the person’s respiratory capacity. While central nervous system activity might result from respiratory, olfactory and/or transdermal delivery, a limited evidence base suggests that topical application achieves much higher plasma levels of linalool and linalyl acetate than inhalation (nothing appears to be known of olfactory delivery) [11]. Known volumes of oil can be applied to the skin for specified periods to readily accessible and relatively absorbent areas [21], leaving residents free to move about as they please. Treatment will fail, however, if the quantities applied are too small to be effective, regardless of mode of administration. When applied topically, lavender is typically prescribed in Australia as a 2.5% lotion but there is no evidence to support this choice. Doses should therefore be substantial so that treatment effects are not missed.

The current study was designed to address these methodological issues by means of a single-blinded, randomised, controlled cross-over trial of high strength, certified pure lavender oil with proven neurophysiological activity. It was administered topically to nursing home residents with significant, persistent physically agitated behaviours that were counted by trained observers before and after application. Physically agitated behaviours were chosen in preference to physically aggressive and verbally agitated behaviours because of their higher frequency and greater ease of measurement. It was hypothesised that lavender would be associated with a significantly greater reduction in selected behaviours than a neutral control oil. Since lavender appears to act as a sedative, its calming effects on behaviour should be accompanied by an evident improvement in emotional well-being. Measures of positive and negative affect were therefore added as outcomes.

## Methods

### Participants

Study participants were recruited from eight specialist psychogeriatric nursing homes and three private nursing homes in Melbourne, Australia, between 2009 and 2011. The following criteria were required for inclusion: (i) a rating by nursing staff of at least mild dementia on the Clinical Dementia Rating scale [22]; (ii) a physically agitated behaviour that occurred at least several times each day in daylight hours, at times other than during nursing care, to a degree that required staff intervention; (iii) an assessment by the nursing staff, visiting medical practitioner and/or psychiatrist that the behaviour was not due primarily to pain, physical illness, depression or psychosis; (iv) residence in the facility for at least three months, and (v) consent to study participation by the next of kin or guardian. Exclusion criteria included: (i) an acute, life-threatening illness; (ii) a variable psychotropic medication regime, and (iii) a medical condition that precluded the use of topical oils. No attempt was made to distinguish one type of dementia from another in this severely disabled group.

The trial was approved by all relevant committees including the Monash University Human Research Ethics Committee. Written consent was obtained from the next of kin or legal guardians of all participants.

### Materials

Four samples of 100% pure lavender oil (*Lavandula angustifolia*) were submitted for electrophysiological analysis to the Department of Pharmacology and Toxicology, Otago University, New Zealand, using established methods [3]. A sample sourced from the Ukraine out-performed others in reducing synaptic activity and membrane excitability in cultured rat embryo pyramidal cells and was therefore selected for use in this trial (Prof. George Lees, personal communication). On preliminary testing, 1 ml of 30% lavender in jojoba oil proved to be the maximum feasible volume and concentration that could be delivered topically. Jojoba oil, which had no electrophysiological activity, was used as a control.

### Study design

The full protocol of this randomized, placebo-controlled, single-blind cross-over trial has been described previously [23]. Participants were allocated randomly by the project manager using an Excel random number generator to either a lavender or control study condition with no pre-set blocking. They were later crossed over to the other condition. To capture any cumulative effects, each of the two experimental conditions comprised three exposures over a one week period with a four-day washout period between them. Treatments were administered at times when nursing staff reported that the selected physically agitated behaviour was most likely to be present, excluding times of personal nursing care. To minimise confounding by intercurrent illness and medication changes, the trial was limited to a two-week period and nursing and medical staff were asked not to alter participants’ psychotropic medications if possible.

The lavender and control oils were stored in identical vials, marked as A or B. Only a single researcher, who had no other involvement in the study, was aware of the allocation. A nursing staff member then massaged 1 ml of either the lavender or control oil into both forearms for one minute each, giving a total of 2 mls per session. Since lavender plasma levels peak after 20 minutes, and are barely detectable after 90 minutes, participants were observed for 30 minutes before and 60 minutes after application [1]. To maintain observer blinding, nurses applying the oil wore a nose clip and research assistants, who completed the observations, applied a mixture of essential oils to their upper lip to disguise lavender’s fragrance. It was not considered practicable or desirable to attempt to blind participants, all of whom had marked cognitive impairment, to the treatment condition.

### Measures

A discretely positioned research assistant recorded if the selected target behaviour was present (one point) or absent (zero points) during each minute giving a maximum score of one point per minute of observation. Results were computed as mean counts over the three 30-minute observation periods. Inter-rater reliability was high with an average Kappa of 0.98 over five training sessions. This time-sampling method has been used successfully in our previous studies [24].

Observers also used the Philadelphia Geriatric Center Affect Rating Scale to record the predominant type of affect (pleasure, contentment, interest, anger, sadness, anxiety/ fear) evident during every minute [25]. The predominant affect was rated as one and all others were rated zero giving a maximum score of one point per minute of observation. Results were averaged as described above. The mean Kappa for inter-rater reliability was 0.92. For reasons of brevity, results for pleasure, contentment and interest are categorized here as positive affects and those for anger, sadness and anxiety/fear as negative affects.

Other measures included the Mini-Mental State Examination (MMSE) and the Cohen-Mansfield Agitation Inventory (CMAI) [26,27]. To identify a suitable behaviour for use as an outcome measure, the CMAI was completed at baseline by nursing staff who rated physically agitated behaviours (e.g. pacing) on a seven-point scale ranging from “occurs never” to “occurs several times an hour” in the previous fortnight.

### Analysis

Sample size was calculated with respect to the primary outcome measure of physically agitated behaviour for a two-sided hypothesis test with a Type I error rate of 0.05 and power set at 90%, based on data from an earlier repeated measures study of simulated family presence versus music therapy in a similar population and setting [24]. In that study, simulated family presence had an effect size of 0.45 relative to the control condition. However, given the mixed evidence supporting lavender, it seemed prudent to apply the smaller effect size of 0.32 for music therapy. It was estimated that 77 participants might be required to detect a difference of this magnitude. At the same time, there were 18 data points for every participant (6 applications x 3 summary behaviour and affect counts per application) which generated considerable statistical power for only a limited number of analyses.

The behaviour and affect scores were over-dispersed relative to Poisson expectations and were therefore modelled using random effects negative binomial regression, taking account of participants’ age, gender and dementia severity. The results of the analyses are shown as mean scores and incidence rate ratios for completed cases. Ratios over one signify an increase in a mean value, and ratios below one signify a decrease in that value, with respect to a reference condition. Ratios are displayed for treatment (lavender versus the control reference condition), time (post-exposure versus the pre-exposure reference condition) and treatment-time interaction.

## Results

**Figure 1:**
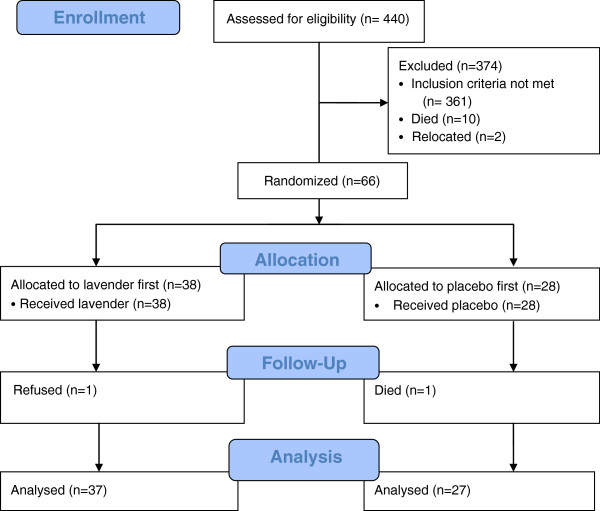
Study Flow Chart.

Figure 1 shows the processes of participant selection, enrolment and follow-up. Finding 66 people suitable for randomization entailed screening 440 residents, most of whom failed to demonstrate sufficient behavioural disturbance. Once the trial began, all but two participants completed both study conditions.

Most participants were female (59%); very old (mean age 77.6 years, S.D. 9.4); markedly cognitively impaired (mean MMSE score 6.1, S.D. 7.0), and physically agitated (mean CMAI score 40.3, S.D. 18.8). The behaviours selected for observation (with numbers) included pacing (55), repetitive mannerisms (5), general restlessness (2), disrobing (1) and intrusiveness (1).

**Figure 2:**
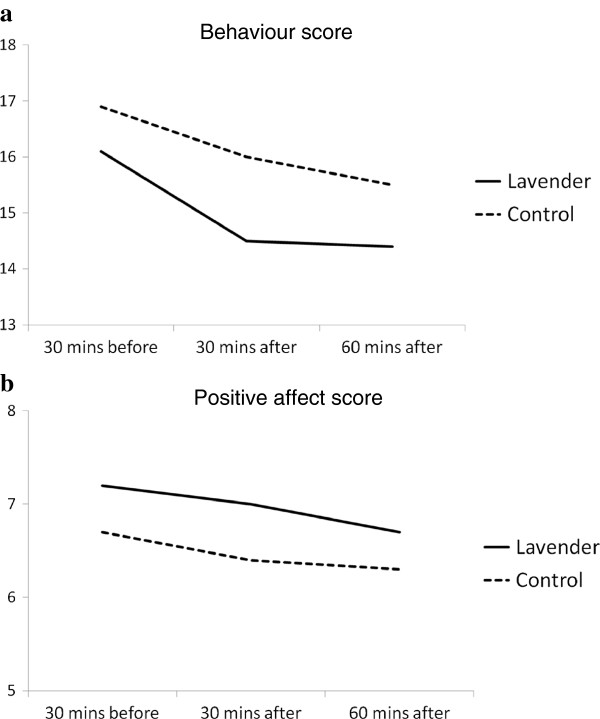
**Mean scores before and after exposure to lavender and control oils. (a)** Behaviour. **(b)** Positive Affect.

For reasons of clarity, raw results are listed in Table 1 and shown graphically in Figures 2a and 2b. Findings of the binomial regression analyses are presented in Table 2.

**Table 1:** Mean (SD) behaviour and affect scores before and after exposure to lavender and control oils

	**Behaviour**	**Affect**	
		**Positive**	**Negative**
**Pre- exposure**			
Lavender	16.1 (10.6)	7.2 (10.2)	0.9 (3.5)
Control	16.9 (10.0)	6.7 (9.7)	1.2 (4.5)
**First 30 minutes post-exposure**			
Lavender	14.5 (10.8)	7.0 (10.1)	0.9 (3.9)
Control	16.0 (10.4)	6.4 (9.9)	1.0 (3.9)
**Second 30 minutes post-exposure**			
Lavender	14.4 (10.6)	6.7 (10.2)	0.7 (3.9)
Control	15.5 (10.7)	6.3 (9.6)	0.9 (3.8)

**Table 2:** Incidence Rate Ratios (IRR) with 95% confidence intervals for effects of treatment, time and treatment-time interaction on behaviour and affect scores after taking account of age, gender and dementia severity

		**Behaviour**		**Affect**			
				**Positive**		**Negative**	
		**IRR**	***p *value**	**IRR**	***p *value**	**IRR**	***p *value**
**Treatment**	Lavender compared to control	0.884 (0.778-1.004)	0.057	1.072 (0.848-1.355)	0.56	0.891 (0.504-1.573)	0.690
**Time**	First 30 minutes post-exposure compared to pre- exposure	0.899 (0.793-1.020)	0.097	0.900 (0.706-1.147)	0.393	0.960 (0.550-1.675)	0.887
	Second 30 minutes post-exposure compared to pre- exposure	0.858 (0.755-0.974)	0.018	0.865 (0.678-1.106)	0.248	0.641 (0.348-1.179)	0.153
**Treatment- time interactions**	Lavender x first 30 min. post-exposure	0.961 (0.798-1.157)	0.672	1.020 (0.726-1.433)	0.910	0.848 (0.371-1.938)	0.696
	Lavender x second 30 min post-exposure	1.045 (0.869-1.259)	0.636	0.954 (0.675-1.348)	0.790	0.687 (0.269-1.750)	0.431

Table 1 and Figure 2a show that agitated behaviour counts were lower in the lavender condition than in the control condition. While this difference approached statistical significance (p = 0.057), the lower counts were evident prior to exposure to lavender, pointing away from a true treatment effect. Similarly, behavior counts fell with time to a statistically significant degree but this occurred regardless of condition. There were no significant treatment-time interactions.

Results were similar for affect ratings (Table 1, Figure 2b). Counts of positive affect were higher in all lavender than control periods but not to significantly different degree. Counts then fell with time, regardless of treatment condition, but this difference was also not significant.

No dermatological or other possible or probable adverse effects were noted as a result of exposure to either the lavender or control oils.

## Discussion

Counts of agitated behaviours were lower during exposure to lavender than to the control oil but this occurred before and after exposure. The observed differences were most probably due to chance. Counts of agitated behaviours also declined over the 90-minute observation period, irrespective of experimental condition, consistent with a spontaneous remission in symptom severity. Treatment was delivered at the time of day when agitated behaviours were expected to peak to maximise the chances of identifying any benefit. Since symptoms wax and wane over the 24-hour period, behaviours were likely to remit regardless of intervention, representing a simple regression to the mean rather than a genuine therapeutic effect.

In devising the protocol, we had sought to avoid the methodological pitfalls identified in previous systematic reviews of treatments of the behavioural and psychological symptoms of dementia [19,28]. Participants had proven, frequent behavioural symptoms; high strength lavender oil with demonstrable neurophysiological activity was administered in a uniform fashion to nursing home residents who were reasonably typical of those with marked behavioural disturbances, and behaviour and affect were measured reliably over a plausible time frame by raters who were blinded to treatment condition.

To ensure statistical power, we sought 77 subjects based on data from an earlier controlled trial of a psychosocial intervention using identical methods in a similar clinical sample. We succeeded in recruiting and retaining only 64 people, despite doubling the study period from one to two years. It seems very unlikely, however, that our negative results are due to inadequate power as there is no suggestion in the raw data of *any* improvement in behaviour or affect during exposure to lavender relative to the control oil based on a limited number of analyses of a very large number of data points.

The most significant limitation to research of essential oils concerns product variability and the limited knowledge base of lavender’s pharmacological properties. There are very few studies of its absorption, metabolism and central nervous system availability in animals and healthy humans, let alone frail older people with dementia. Lavender appears to be well absorbed and to have sedative and anticonvulsant potential but information is otherwise limited with the result that dosing strategies are based largely on traditional practice. We settled on a topical dose of 1 ml 30% certified pure lavender oil applied to each forearm only because a higher strength was so viscous that application became impracticable. This was still much higher than the doses delivered by vapour in most previous studies. Future researchers should consider measuring plasma levels of linalool and linalyl acetate as a check on absorption.

The current lack of information also makes it difficult to understand why two studies have returned positive results while five others, including the present one, have not. Without a consistent proprietary formulation and sound pharmacokinetic data, of the kind now reported routinely for new pharmaceutical products, there may be little value in further clinical trials.

## Conclusion

Studies of essential oils are constrained by their variable formulations and uncertain pharmacokinetics and so optimal dosing and delivery regimens remain speculative. Notwithstanding this, topically delivered, high strength, pure lavender oil had no discernible effect on affect and behaviour in a well-defined clinical sample.

## Competing interests

The authors do not have competing interests.

## Authors’ contributions

All the authors participated in the preparation of the manuscript and have read and approved the final draft. DO’C conceptualised and designed the study; EvdP and BE conducted the study and analysed the results, and JT helped with statistical analysis. All authors have read and approved the manuscript.

## Pre-publication history

The pre-publication history for this paper can be accessed here:

http://www.biomedcentral.com/1472-6882/13/315/prepub
